# Tumor necrosis factor-mediated inhibition of interleukin-18 in the brain: a clinical and experimental study in head-injured patients and in a murine model of closed head injury.

**DOI:** 10.1186/1742-2094-1-13

**Published:** 2004-07-28

**Authors:** Oliver I Schmidt, Maria Cristina Morganti-Kossmann, Christoph E Heyde, Daniel Perez, Ido Yatsiv, Esther Shohami, Wolfgang Ertel, Philip F Stahel

**Affiliations:** 1 Department of Trauma and Reconstructive Surgery, Charité University Medical School, Campus Benjamin Franklin, Berlin, Germany; 2 Department of Trauma Surgery, The Alfred Hospital, Monash University, Melbourne, Australia; 3 Department of Surgery, University Hospital Zurich, Switzerland; 4 Department of Pharmacology, The Hebrew University, Hadassah Medical School, Jerusalem, Israel

**Keywords:** Closed head injury, inflammation, cytokines, TNF, interleukin-18.

## Abstract

Tumor necrosis factor (TNF) and interleukin-(IL)-18 are important mediators of neuroinflammation after closed head injury (CHI). Both mediators have been previously found to be significantly elevated in the intracranial compartment after brain injury, both in patients as well as in experimental model systems. However, the interrelation and regulation of these crucial cytokines within the injured brain has not yet been investigated. The present study was designed to assess a potential regulation of intracranial IL-18 levels by TNF based on a clinical study in head-injured patients and an experimental model in mice. In the first part, we investigated the interrelationship between the daily TNF and IL-18 cerebrospinal fluid levels in 10 patients with severe CHI for up to 14 days after trauma. In the second part of the study, the potential TNF-dependent regulation of intracerebral IL-18 levels was further characterized in an experimental set-up in mice: *(1) *in a standardized model of CHI in TNF/lymphotoxin-α gene-deficient mice and wild-type (WT) littermates, and *(2) *by intracerebro-ventricular injection of mouse recombinant TNF in WT C57BL/6 mice. The results demonstrate an inverse correlation of intrathecal TNF and IL-18 levels in head-injured patients and a TNF-dependent inhibition of IL-18 after intracerebral injection in mice. These findings imply a potential new anti-inflammatory mechanism of TNF by attenuation of IL-18, thus confirming the proposed "dual" function of this cytokine in the pathophysiology of traumatic brain injury.

## Findings

Closed head injury (CHI) is the leading cause of mortality and persisting neurological impairment in young people in industrialized countries [1,2]. The neuropathological sequelae of brain injury are mediated in large part by a profound host-mediated intracranial inflammatory response [3-5]. The pro-inflammatory cytokines tumor necrosis factor (TNF) and interleukin (IL)-18 have been identified as crucial mediators of neuroinflammation after brain injury [6-9]. This notion has been supported by experimental studies in rodents which demonstrated neuroprotective effects by pharmacological inhibition of either TNF or IL-18 after CHI [9-11]. In recent years, the concept of a "dual role" evolved with regard to concomitant beneficial and adverse effects of pro-inflammatory mediators, depending on the kinetic of their expression and posttraumatic regulation in the injured brain [3,12,13]. However, the TNF-dependent regulation of IL-18 in the injured brain has not yet been investigated. We sought to determine the interrelationship between intracranial TNF and IL-18 levels in a clinical study on patients with severe CHI and in an experimental model in mice.

Patients with isolated severe closed head injury (*n *= 10, Glasgow Coma Scale score ≤ 8) and indication for intraventricular catheters for cerebrospinal fluid (CSF) drainage due to increased intracranial pressure (ICP > 15 mm Hg) were included in this study. Drained CSF was collected daily for up to 14 days after trauma or until catheters were removed. The patient characteristics are shown in Table 1. No patient was treated with steroids. The protocol for daily CSF collection is in compliance with the Helsinki Declaration and was approved by the University's Ethics Board Committee. Control CSF was collected from patients undergoing diagnostic spinal tap (*n *= 10) and revealed no inflammatory CNS disease, based on normal CSF protein and glucose levels and normal white cell counts. All samples were kept on ice at 4°C and immediately centrifuged after collection, aliquoted, and frozen at -70°C until analysis.

**Table 1:** Clinical data and intrathecal cytokine levels in patients with severe closed head injury.

**Patient**	**Age (years) / Gender**	**Type of brain injury (Marshall score)**	**Outcome (GOS)**	**TNF in CSF (pg/mL)**		**IL-18 in CSF (pg/mL)**		**Correlation *r***_S_
				**Mean**	**Range**	**Mean**	**Range**	

**1**	38 / M	EML	4	6.4	1.0 – 11.5	40.6	6.5 – 155.2	- 0.804 **
**2**	30 / M	DI II°	3	3.6	1.0 – 7.7	114.3	29.7 – 286.4	- 0.580 *
**3**	56 / M	EML	4	6.3	1.0 – 10.0	35.1	11.2 – 100.3	- 0.530
**4**	57 / F	DI II°	5	6.0	1.0 – 11.7	20.1	5.0 – 168.8	- 0.761 **
**5**	44 / M	EML	4	1.6	1.0 – 3.4	39.8	22.6 – 74.5	- 0.751 *
**6**	26 / M	EML	4	3.2	1.0 – 10.3	108.5	5.0 – 328.6	- 0.832 **
**7**	47 / M	EML	1	1.1	1.0 – 1.4	268.5	78.3 – 462.0	- 0.372
**8**	25 / M	EML	4	2.2	1.0 – 4.0	91.6	10.3 – 290.0	- 0.195
**9**	37 / F	DI III°	3	1.6	1.0 – 2.7	183.7	21.5 – 382.2	- 0.844 **
**10**	35 / M	DI II°	4	2.0	1.0 – 5.8	209.4	19.9 – 391.8	- 0.772 *
**Controls**	**(*n *= 10)**			1.0	1.0 – 7.1	5.0	5.0 – 8.4	

Statistical analysis for assessment of the correlation between tumor necrosis factor (TNF) and interleukin (IL)-18 levels in serial cerebrospinal fluid samples for up to 14 days after trauma was performed by Spearman's rank correlation (*P < 0.05, **P < 0.01). The patients' outcome was determined at 3 months after injury by the Glasgow Outcome Scale (GOS) score [33]: 5 = asymptomatic, 4 = moderate disability, 3 = severe disability, 2 = persisting vegetative state, 1 = death. The type of brain injury was classified by the CT-scan criteria established by Marshall et al. [34] into diffuse injury (DI) grade I-III and evacuated *vs. *non-evacuated mass lesions (EML, NEML).

Quantification of IL-18 and TNF levels in human CSF samples and in murine brain homogenates was performed by species-specific commercially available ELISA (R&D Systems, Abingdon, UK). According to the information provided by the manufacturer, the IL-18 assay recognizes both the mature and the pro-form of IL-18. All concentrations below the detection limit of 5 pg/mL (IL-18) or 1 pg/ml (TNF) were assigned a value of 5 pg/mL, and 1 pg/ml respectively. All samples were run undiluted in duplicate wells. The concentrations were calculated from the mean OD of duplicate samples, determined by spectrophotometer (Dynatech Laboratories Inc., Chantilly, VA, U.S.A.) at an extinction wavelength of 405 nm.

The experimental part of the study was set-up on two different protocols with the aim to assess the TNF-dependent regulation of IL-18 in the murine brain:

*(1) *The first part of the experimental study was designed to investigate a potential role of TNF-dependent regulation of intracranial IL-18 expression in a standardized model of CHI, using mice double-deficient in genes for TNF and lymphotoxin-α (TNF/LT-α-/-) [14]. These knockout mice were selected in order to compensate for potential redundant functions of TNF by LT-α which binds to the identical common receptors (i.e. TNF receptors p55 and p75) [14-16]. The generation and development of the TNF/LT-α-/- mice on a mixed C57BL/6 × 129Sv/Ev (B6 × 129) genetic background has been previously described [14]. Knockout mice and wild-type (WT) littermates of the B6 × 129 strain were subjected to a CHI (*n *= 10 per group) using a standardized weight-drop model, as previously described [9,17]. In brief, following ether anesthesia, a midline longitudinal scalp incision was performed. Trauma was applied to the left anterior frontal area of the exposed skull by a 330 g weight with a silicon tip dropped from a height of 2 cm, resulting in a focal closed injury to the left hemisphere. Mice received supporting oxygenation with 100% O_2 _until they awakened and were then brought back to their cages. Control animals were subjected to anesthesia and sham operation only (*n *= 10 per group). In addition, mice with anesthesia alone (*n *= 8) were used as internal control and untreated control animals (*n *= 10) were analyzed for baseline evaluation of intracerebral cytokine profiles in these mice.

*(2) *In the 2^nd ^part of the experimental study, mice of the C57BL/6 strain (*n *= 10 per group) were treated by stereotactic intracerebroventricular (i.c.v.) injection of either 200 ng mouse recombinant TNF in 10 μl PBS, or vehicle solution only (10 μl PBS), into the left hemisphere using a sterile 27-gauge syringe, under ether anesthesia. According to data from previously published studies [18-20], as well as based on titration studies from our own laboratory, the i.c.v. injection of 200 ng mouse-recombinant TNF (R&D Systems) elicited an evident induction of inflammatory changes in the murine CNS, such as intracranial recruitment of leukocytes and development of brain edema in the injected hemisphere within 24 hours (data not shown). Animals from all groups (CHI and i.c.v. injection) were sacrificed at 24 h after the respective procedure, which corresponds to the time-point of maximal extent of intracerebral inflammation in the model of CHI used in this study [17].

For assessment of intracerebral IL-18 levels, the murine brains were immediately removed after decapitation. Tissue homogenization was performed using a Polytron homogenizer (Kinematica, Kriens, Switzerland) with a dilution of 1:4 in ice cold extraction buffer containing 50 mmol/L Tris buffer (pH 7.2), NaCl 150 mmol/L, Triton-X-100 1%, and protease inhibitor cocktail (Roche, Mannheim, Germany). The homogenates were shaken on ice for 90 minutes, centrifuged for 15 minutes at 3,000 g and 4°C. Thereafter, the supernatants were aliquoted and stored at -70°C until analysis. The concentrations of total protein in the brain extracts were measured by Bradford assay (Bio Rad Laboratories, Munich, Germany). The intracerebral protein concentrations were in a similar range in all mice assessed (11.7 ± 2.4 mg/mL; mean ± SD). The quantification of IL-18 levels in murine brain homogenates was performed as described above for the human samples. All mice used in this study were males, in order to avoid a bias in gender, age 12 to 16 weeks with body weights of 25 to 32 g. The animal experiments were performed in compliance with the guidelines of the Federation of European Laboratory Animal Science Association (FELASA) and approval was granted by the Institutional Animal Care Committee of the University of Zurich and of the Hebrew University of Jerusalem.

In the clinical part of the study on CHI patients, the mean IL-18 concentrations in ventricular CSF collected up to 14 days after trauma were significantly higher than in control lumbar CSF from patients undergoing diagnostic spinal tap (*P*< 0.05, repeated measures ANOVA; Table 1). These findings are coherent with data from previously published studies which demonstrated significantly elevated intracranial IL-18 levels after brain injury, both in humans as well as in experimental model systems [8,9]. With regard to TNF, the mean levels in individual serial cerebrospinal fluid samples were significantly elevated in 50% of all head-injured patients, compared to controls (patients #1,2,3,4,6; Table 1). Elevated intracranial TNF levels after traumatic brain injury have been previously reported in various clinical and experimental studies [21-26]. In the present study, the individual daily TNF levels in CSF were up to 10- to 100-fold lower than the corresponding IL-18 levels (Table 1). Interestingly, despite the fairly low TNF levels in CSF we found an inverse correlation between the daily individual intrathecal TNF and IL-18 levels in all trauma patients, as demonstrated by a negative Spearman's rank correlation coefficient (*r *= -0.195 to -0.844). In 7 of 10 patients, this inverse correlation was statistically significant, with a *P*-value < 0.05 in three patients (# 2,5,10) and *P *< 0.01 in four patients (# 1,4,6,9; Table 1). Since the quality of the blood-brain barrier has not been determined in this cohort of head-injured patients, due to the lack of matching serum samples for assessment of albumin levels, the source of elevated cytokines (intrathecal compartment *vs. *peripheral serum) remains unclear.

**Figure 1:**
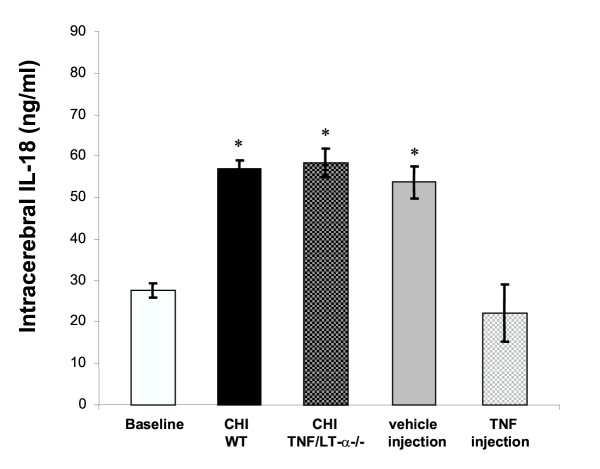
Intracerebral IL-18 concentrations in mice, as determined by ELISA in murine brain homogenates (*n *= 10 animals per group). Untreated normal mice were used for determination of baseline IL-18 levels in this study. The four treatment groups were sacrificed after 24 hours for assessment of intracerebral IL-18 levels, as described in the text. Mice deficient in genes for TNF and lymphotoxin-α (TNF/LT-α-/-) and wild-type (WT) littermates were subjected to focal closed head injury (CHI) and sacrificed after 24 hours. Two additional groups of WT mice were given an intracerebro-ventricular (i.c.v.) injection of 200 ng mouse-recombinant TNF in 10 μl PBS or by vehicle alone (10 μl PBS i.c.v.). The data are presented as means ± SEM. *P < 0.01 vs. baseline / TNF-injection (unpaired Student's *t*-test).

In the experimental study, IL-18 was found to be constitutively expressed in the brain of untreated mice (27.7 ± 5.4 ng/mL, mean ± SEM; "baseline", Fig. 1), which is in accordance with data from previous studies revealing constitutive IL-18 expression in the CNS of normal rats and mice [8,9,27,28]. Microglia may represent the cellular source of constitutive intracranial IL-18 levels in these mice, since Prinz and colleagues have previously shown that microglial cells, but not astrocytes, produce IL-18 in the murine CNS [28]. The intracerebral IL-18 levels increased significantly in the head-injured group by 24 hours after experimental CHI (56.9 ± 4.7 ng/mL, *P *< 0.01 *vs. *baseline, Fig. 1). Knockout mice lacking TNF and LT-α genes also showed significantly elevated IL-18 concentrations at 24 h after CHI (58.4 ± 7.8 ng/mL) which were in a similar range as in head-injured WT mice, as shown in Fig. 1. This lack of differences in mice deficient in the ligands for TNF receptors may be explained by alternatively expressed inflammatory mediators or modified pathways of IL-18 regulation in these genetically engineered mice which have been shown to have significantly altered immune responses [14]. Interestingly, the intracerebral injection of vehicle alone (10 μl PBS i.c.v.) induced significantly elevated IL-18 levels in brains of normal WT mice, compared to normal untreated mice, which were in a similar range as in the brain-injured groups (53.6 ± 9.6 ng/ml, Fig. 1). These data imply that a minor penetrating injury by i.c.v. injection of a small volume of buffer solution represents a procedure which is sensitive enough to induce significant IL-18 production in murine brains within 24 hours. In contrast, the intracerebral injection of murine recombinant TNF (in 10 μl PBS) reduced the elevated IL-18 levels in murine brains significantly to levels than were even lower than baseline concentrations (22.13 ± 7.1, *P *< 0.01 *vs. *vehicle-injected mice), as shown in Fig. 1.

The findings from these experimental investigations corroborate the data from the clinical study, where an inverse correlation of intrathecal TNF and IL-18 levels during the first 14 days after severe CHI was found, suggesting that the inhibition of IL-18 may represent a new potential anti-inflammatory mechanism after CHI. Such a "dual role" of TNF has been suggested previously in terms of concomitant pro- and anti-inflammatory effects and detrimental as well as beneficial neuroprotective properties after brain injury [12]. While the pro-inflammatory effects mediated by TNF in the CNS have been thoroughly investigated in the past two decades [15,29,30], the concept of anti-inflammatory effects mediated by cytokines which have been formerly designated as "pro-"inflammatory mediators, is still challenging and novel. Scherbel and colleagues were the first to provide evidence of beneficial effects of TNF in the later phase (i.e. 4 weeks) after traumatic brain injury, based on studies in TNF-/- mice subjected to controlled cortical impact brain injury [31]. In these experiments, the TNF-deficient mice showed a significantly attenuated neurobehavioral impairment than WT littermates in the first 48 hours post trauma, in terms of early detrimental effects mediated by TNF [31]. However, the expected neurobehavioral recovery was absent in TNF-/- mice after 4 weeks and cortical tissue loss was significantly increased at this time-point, compared to WT littermates, implying that at later time-points the lack of TNF leads to adverse outcome after brain injury [31]. Barger and colleagues have previously shown in an *in vitro *model of amyloid β-mediated neurotoxicity that both TNF and LT-α (formerly designated TNF-β) can significantly attenuate neuronal degeneration by induction of antioxidative pathways through activation of the transcription factor NFκb, thus strengthening the notion of neuroprotective effects mediated by these cytokines [32]. This assumption was further corroborated in the setting of experimental CHI, where mice lacking both TNF and LT-α genes were shown to have a significantly increased mortality within one week after trauma, compared to WT littermates [17]. Overall, these data support the notion that TNF exerts detrimental effects in the early phase and beneficial neuroprotective effects in the later phase after head injury [12]. However, the assumptive underlying regulatory mechanisms of TNF-mediated neuroprotection and of TNF-mediated suppression of IL-18 in the injured brain remain unclear and have to be investigated in future experimental studies.

## List of abbreviations

Central nervous system (CNS), cerebrospinal fluid (CSF), closed head injury (CHI), intracerebroventricular (i.c.v.), interleukin (IL), lymphotoxin-α (LT-α / TNF-β), nuclear factor κB (NFκB), tumor necrosis factor (TNF), phosphate-buffered saline (PBS), intracranial pressure (ICP), wild-type (WT), enzyme-linked immunosorbent assay (ELISA).

## Competing interests

There are no financial interests by any of the authors with regard to the present project.

## Authors' contributions

OIS, MCMK, CEH, ES, and PFS were responsible for conception and planning of the experiments, as well as for performing the animal experiments, collection of the human cerebrospinal fluid samples and cytokine measurements in human and murine tissue samples, as well as for writing of the manuscript. DP and IY performed the experimental i.c.v. injection experiments. WE contributed to the interpretation of the results and writing of the manuscript.
